# A database of PCR primers for the chloroplast genomes of higher plants

**DOI:** 10.1186/1746-4811-3-4

**Published:** 2007-02-27

**Authors:** Berthold Heinze

**Affiliations:** 1 Department of Genetics, Federal Research Centre for Forests, Hauptstrasse 7, Vienna, Austria

## Abstract

**Background:**

Chloroplast genomes evolve slowly and many primers for PCR amplification and analysis of chloroplast sequences can be used across a wide array of genera. In some cases 'universal' primers have been designed for the purpose of working across species boundaries. However, the essential information on these primer sequences is scattered throughout the literature.

**Results:**

A database is presented here which assembles published primer information for chloroplast DNA. Additional primers were designed to fill gaps where little or no primer information could be found. Amplicons are either the genes themselves (typically useful in studies of sequence variation in higher-order phylogeny) or they are spacers, introns, and intergenic regions (for studies of phylogeographic patterns within and among species). The current list of 'generic' primers consists of more than 700 sequences. Wherever possible, we give the locations of the primers in the thirteen fully sequenced chloroplast genomes (*Nicotiana tabacum, Atropa belladonna, Spinacia oleracea, Arabidopsis thaliana, Populus trichocarpa, Oryza sativa, Pinus thunbergii, Marchantia polymorpha, Zea mays, Oenothera elata, Acorus calamus, Eucalyptus globulus, Medicago trunculata*).

**Conclusion:**

The database described here is designed to serve as a resource for researchers who are venturing into the study of poorly described chloroplast genomes, whether for large- or small-scale DNA sequencing projects, to study molecular variation or to investigate chloroplast evolution.

## Background

In 1991, Pierre Taberlet published what was probably the first article recommending 'universal' polymerase chain reaction (PCR) primers for use across plant genera and species, with a view to analysing intra-specific variation [1]. The approach has been favourably adopted by the scientific community: a recent search identified 855 citing papers for the original publication (Scholar Google, 25 October 2006; up from 678 in January 2006). New sets of primers have subsequently been published that reflect Taberlet's original intention to study molecular variation among closely related species, or among separate sets of populations within species, by analysing introns and spacers [e.g., [2-5]]. This can be done since chloroplast genes evolve slowly, and primers can be designed with the purpose of working across species. In chloroplast genomes, gene order is highly conserved [2,3,5], whereas some spacers show even intra-species variation. Amplified fragments can be analysed by restriction analysis or DNA sequencing. The author's experience is that small insertions/deletions (indels) are relatively frequent, when compared to point mutations that result in restriction site changes [6-8]. Exon sequences are generally highly conserved, but this depends on the gene in question. Molecular systematicists, starting with the highly conserved *rbcL *gene, and later expanding to e.g. *matK*, *ndhF*, *rpl16*, and *atpB*, have utilized PCR-amplified chloroplast gene sequences for establishing and verifying phylogenies. Sets of primers recommended for this purpose have also expanded in size [e.g., [9-12]]. We have published a core set of 38 primer pairs useful in the amplification of the large single copy region in angiosperms, but also for fragments of this region in other plants [13].

As more and more partial and complete chloroplast DNA genome sequences become available, it is apparent that a balanced view on chloroplast sequence variation depends on the choice of many different sites along the genome [10,11]. It is interesting to notice that different groups of authors tend to work with alternative sets of primers. A central site for primer information should therefore help in making resources that are already there more widely known, and to encourage comparative studies across many laboratories.

## Construction and content

### Published primer sequences

**Figure 1:**
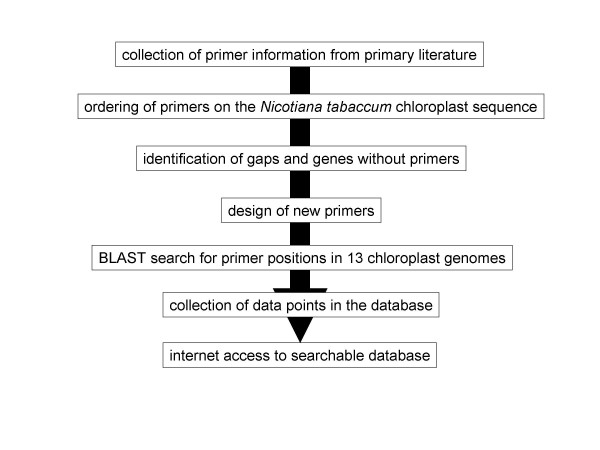
Overall scheme of construction and content of the database.

An overall scheme on the construction of the database is given in Figure 1. Published articles were screened in a random fashion for new primer information between 1999 and 2005. This included scanning the tables of contents of the following journals manually: *American Journal of Botany, Annals of Botany, Belgian Journal of Botany, Biochemical Systematics and Ecology, Biologia Plantarum, Canadian Journal of Botany, Conservation Genetics, Genetic Resources and Crop Evolution, Heredity, Molecular Biology and Evolution, Molecular Breeding, Molecular Ecology, Molecular Ecology Notes, Molecular and General Genetics, Molecular Phylogenetics and Evolution, New Phytologist, Plant Molecular Biology, Plant Molecular Biology Reporter, Plant Science, Plant Systematics and Evolution, Planta, Sexual Plant Reproduction, Systematic Botany, Theoretical and Applied Genetics*, and *Trees Structure and Function*. Among those, *Molecular Ecology Notes *contains a section of its own, devoted to primer information. Furthermore, the following literature databases were searched, using the key words 'chloroplast', 'PCR', and 'primer' in titles, abstracts and key words: Current Contents Connect [14], Scopus [15], and Forest Science Info [16]. While it is not easily possible to check each individual article from the vast body of scientific literature for descriptions of chloroplast DNA variation, it is also tedious to check those with the relevant keywords for new primer information, as this is often not even mentioned in the abstract. In general, new primers for the database were extracted from articles describing more than one primer pair which was anchored in conserved chloroplast regions (i.e., within genes). Published primers cover a large section of known chloroplasts. All sources of primer information (references) are listed online in the database (a list can be generated by sorting the database for the 'References' column). The majority of primers are from articles describing larger sets of primer pairs, typically more than four. A lists of published primers was kindly provided by Bill Hahn (Columbia University, NY, USA) in 1999. Kevin Livingstone (Trinity University, San Antonio, TX, USA) supplied an excerpt from the *Molecular Ecology Notes *database of primers regarding chloroplast-specific entries in 2004. This database is now online [17]. The latest additions are from a paper by Dhingra and Folta [18] covering the whole inverted repeat region.

The primers were initially ordered along the tobacco (*Nicotiana tabaccum*) chloroplast genome (GenBank:Z00044 and S54304), as this is the best characterised chloroplast genome to date. Orientation of the primers (F, forward; R, reverse) is given relative to the tobacco sequence. These manipulations were mainly done with the Omiga 1.1.3 software (Accelrys, Oxford, UK).

### Filling the gaps

Together with Delphine Grivet and Remy Petit (then at INRA-Pierroton, France), primers were designed in order to close any remaining gaps along the large single copy region. As a result of this collaboration, a set of 38 primer pairs spanning this region of the chloroplast genome in angiosperms was published [13]. The primer pairs described in this publication [13] amplify fragments of between 2000 and 5000 bp in most angiosperm species. Methods for identifying conserved primer binding sites included comparing and aligning chloroplast DNA sequences available in GenBank by eye, with the help of Omiga, by using BLAST [19], or by using a suite of DOS programs written by John Antoniw [20,21]. Further primers were developed for this database following the same strategy, in order to fill remaining gaps, to decrease the size of amplicons, or to replace primers with poor performance in our lab. Sufficiently conserved potential primer sites in alignments were visually checked for abnormalities like biased GC/AT percentages, mononucleotide stretches, or apparent palindromes; sites with such features were avoided whenever possible. In some cases, alternative primer sites were designated in close proximity, so that the users can select the ones best matching their taxa of interest.

### Positioning primers in sequenced chloroplast genomes

BLASTALL (obtained from NCBI, USA [19]), was used to search for homologies of the primers in 13 chloroplasts (from GenBank, December 2005, except *Populus*): *Nicotiana tabacum *(GenBank:NC_001879.1), *Atropa belladonna *(GenBank:NC_004561), *Spinacia oleracea *(GenBank:NC_002202.1), *Arabidopsis thaliana *(GenBank:NC_000932), *Populus trichocarpa *[Heinze *et al*. in preparation, and [22]], *Oryza sativa *NC_001320.1, *Pinus thunbergii *(GenBank:NC_001631.1), *Marchantia polymorpha *(GenBank:NC_001319.1), *Zea mays *(GenBank:X86563), *Oenothera elata *(GenBank:AJ2710796.2), *Acorus calamus *(GenBank:NC_007407.1), *Eucalyptus globulus *(GenBank:AY780259.1), and *Medicago trunculata *(GenBank:NC_003119.6) with an E value cut-off of 0.5. The position of the 5' nucleotide for each primer in the 13 full genome sequences is given whenever sufficient homology (E value below 0.5) was found. For primers with multiple binding sites (e.g., those in the inverted repeats), only the position of the first site is given. There are cases where BLAST returned spurious primer binding sites in some of the species (not in the expected position) and these indicate possible sources for PCR artefacts. Therefore, such primer positions are also included in the database. Different weights were not given to matches in the 5' or 3' ends of the primers in the BLAST search, because in a low-complexity template as the plant chloroplast, even sub-optimal priming may lead to amplification (and consequently, artefacts).

With this data set, it is easily possible to calculate PCR fragment sizes, and to estimate expected sizes from 'new' taxa. Primer designations from the original publications were retained as much as possible. Sometimes, the name of the first author or some other hints were included in the primer names, in order to make them unique. 'F' and 'R' are indicating the direction (forward or reverse) of the primer relative to the tobacco sequence (some authors have named their primers on the basis of the direction of transcription, which can be a source of confusion here). 'P' and 'M' denote 'plus' or 'minus' primer directions in a similar way. A few statistics for the content of the current database (version 2.1) are given in Table 1.

**Table 1:** Statistics of database version 2.1

**condition**	number of database entries*
total number of entries	719
anchored in *Nicotiana tabacum*	693
anchored in *Atropa belladonna*	647
anchored in *Spinacia oleracea*	534
anchored in *Arabidopsis thaliana*	535
anchored in *Populus trichocarpa*	540
anchored in *Eucalyptus globulus*	595
anchored in *Medicago trunculata*	495
anchored in *Oenothera elata*	534
anchored in *Zea mays*	512
anchored in *Oryza sativa*	488
anchored in *Acorus*	543
anchored in *Pinus thunbergii*	351
anchored in *Marchantia polymorpha*	263
present in all 13 chloroplast genomes	138
present in *Nicotiana *and *Atropa*	646
present in *Nicotiana*, *Atropa *and *Spinacia*	523
present in *Arabidopsis*, *Populus *and *Eucalyptus*	465
present in all dicots	336
present in monocots	397
present in all 13 but *Pinus *and *Marchantia*	60
present in all 13 but *Marchantia*	52
within *trn *genes	132
within photosystem genes (*psa*, *psb*)	94
within ribosomal proteins and RNA polymerase (*rpl*, *rps*, *rpo*)	120
within ATPase genes (*atp*)	33
within *ycf *genes	46
within *rbcL*	38
within NADH-specific dehydrogenase (*ndh*) genes	23

* Summary results of BLASTALL homology searches (e-value above 0.5) are shown.

### Overview graphics

**Figure 2:**
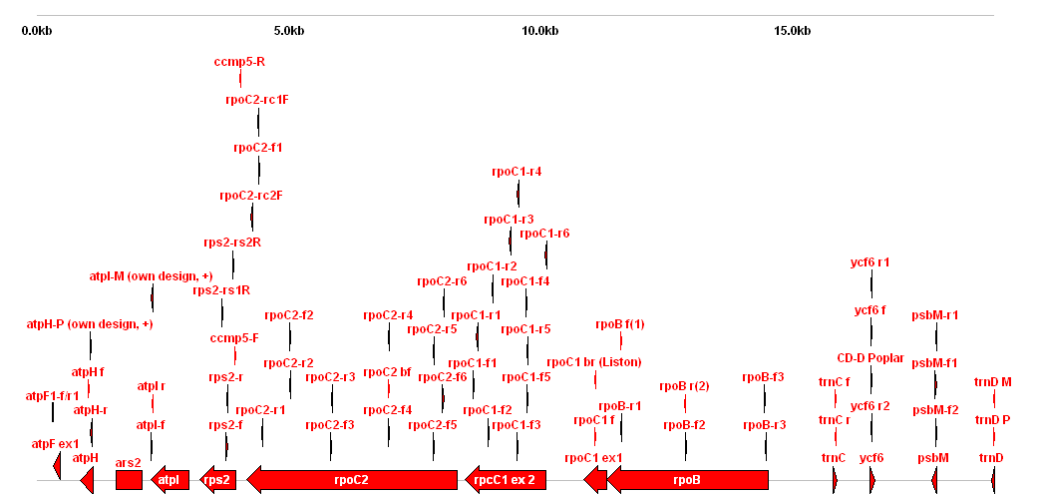
**Primer positions in a section of the tobacco chloroplast**. Bars represent genes, and triangles represent primers, with their respective orientations indicated by the arrows, along a stretch of the tobacco chloroplast genome.

Most of the primers are included as 'features' in a set of Omiga/DS Gene 1.5 (Accelrys, Oxford, UK) sequence files. Graphics were exported from these programmes and are available on the database website. An example is given in Figure 2.

## Utility and Discussion

Transferring PCR primers to new species has accelerated molecular research tremendously. However, it should be noted that from the early days of chloroplast genome research, when probes and blotting techniques were still in use, cross-species transfer of such gene probes was always possible [e.g., [23]]. Nevertheless, venturing into unknown species with primers is still a 'trial and error' experience. It is hoped that the availability of a database that includes alternative, tested primers for a number of species will reduce the efforts in such cases. The database can now easily be searched or filtered: a text field is included which allows for free-text searching in any of the fields. For example, this text may be a part of a gene or primer name, an author name in the references (column 'Ref_Src'), or even a part of a primer nucleotide sequence. Additionally, the data can be sorted for the values in any of the fields. Numerical values (e.g., primer length or position in any of the 13 genomes) will be sorted arithmetically, and text values alphabetically. For instance, a search and filter operation for all primers associated with transfer RNA (trn) genes would require typing 'trn' into the filter; the results can be sorted e.g. by species (by entering the column number 11 for e.g. *Acorus*) or by gene names (column 3). Positioning the mouse over the column headings will show the full name of the columns.

Furthermore, it is possible to combine primers from different publications into newly assembled pairs. 'Generic' PCR conditions that favour successful amplification with such new combinations are standard PCR conditions with the use of 2 mM Mg^2+ ^and a PCR program with 10 cycles at 70°C annealing, followed by 32 cycles at 55°C or 50°C annealing [7]. With these primers and methods, the chloroplast genomes of hitherto unexplored species can be scanned in detail, and regions specific for different purposes picked [e.g. [7,8]].

The author's recommendation for analysing uncharacterized chloroplast genomes is the following: (i) select a genome from the 13 listed which is phylogenetically close to the species of interest; (ii) sort the database for primer position in the selected genome (leaving the 'filter' field empty so that all primers will be displayed); (iii) select primer pairs in a suitable distance in the selected genome. In this last step, care must be taken to select primer pairs in the correct orientation. This can be done by comparing the gene order in the region of interest with the one in tobacco. In case of similar orientation of the genes, the 'F' and 'R' designations of the primers can be used as given in the database. In case of reverse orientation of the local gene order (relative to tobacco) in the species of interest, primer orientation is also reverse.

Recently, Dhingra and Folta [18] have suggested using overlapping PCR fragments for sequencing entire chloroplast genomes from total plant DNA preparations. While this approach holds some promise, it must be mentioned that a number of phenomena can interfere with it. Chloroplast DNA fragments are constantly transferred to the nucleus and to the mitochondrion [24], and extra-chloroplast DNA can lead to artefacts like apparent sequence polymorphisms. We have encountered such polymorphisms in shotgun sequencing of the black poplar (*Populus trichocarpa*) genomes [Heinze *et al*. in preparation; [27]]. The quality of the DNA preparation (relative amounts of nuclear, mitochondrial, and chloroplast DNA) is key to the success of the PCR sequencing strategy. Prior purification of chloroplasts, which has always been a speed-limiting step, will determine the success to a large degree. The advantage of using primers from this database, as opposed to a fixed set of primer pairs, is that it is easy to switch from unsuccessful primers to alternatives, as very often, alternative primer positions close to each other, and degenerate sequences, have been proposed by different authors. It is also easier to generate overlapping sequence when primers are employed in varying combinations.

This version of the database offers the following improvements compared to earlier ones [26]: the database can now be searched, filtered, and sorted online; there are now more than 700 primers (up from 500+); and primer positions are now given in 13 genomes (previously only five). In comparison to the single article introducing the highest number of primers [13], this database contains almost 10 times as many primers.

We have analysed collections of wild cherry (*Prunus avium*) and common ash (*Fraxinus excelsior*) DNAs for variation in chloroplast sequence, using either the PCR-RFLP [25] or a denaturing high-performance liquid chromatography approach [Heinze in preparation; [8,26]]. In both cases, it was possible to quickly screen the major part of the large single copy region for variation between samples collected from different sites across the species ranges. In our laboratory, agarose PCR-RFLP is still a first screening method with acceptable throughput, when large sample numbers are analysed. After PCR, samples are scanned on agarose gels for successful amplification. An aliquot of the PCR is treated with restriction enzymes. Restriction polymorphisms and major indels can be detected in high-percentage agarose gels.

Some problems with universal PCR include the more rapid sequence evolution in some parts of the chloroplast; the identification of polymorphisms between conserved primer sites (introns), and occasional rearrangements, deletions, duplications in some genera, families, or higher taxonomic groups. However, rearrangements often only affect one spacer, leaving large blocks of genes with their order conserved. It is often at the breakpoints between conserved blocks of gene order where more sequence variation at lower taxonomic levels can be found.

It is tempting to speculate about poorly characterised chloroplast DNA regions that nevertheless yield conserved primer binding sites. This happens in some introns, but also in a number of spacers, open reading frames (ORFs) or hypothetical conserved reading frames (ycfs).

## Conclusion

Conserved 'universal' primers and markers are possible for chloroplast DNA. Polymorphisms can be identified with tested primer sequences from the database. 'Generic' PCR conditions make possible the use of many primers in new combinations. Several opportunities exist for efficient detection and analysis of polymorphisms. It is hoped that this database will prove useful for many diverse problems and that our knowledge of mutation and evolution processes in chloroplasts will subsquently be enhanced, making it possible in the future to postulate informed predictions for poorly characterized species. Additions to the database (by e-mail to the author) are welcome.

## Availability and requirements

### Database internet address

The database is currently available at  and at . Along with the description and the full primer table, an Excel file (Additional File 1) is available for downloading and individual sorting or printouts.

## Competing interests

The author(s) declare that they have no competing interests.

## Supplementary Material

Additional file 1 Primers Jan 2007. The data base as a static version as of January 2007, in Microsoft Excel format. Click here for file
